# Introduction of less invasive surfactant administration (LISA), impact on diagnostic and therapeutic procedures in early life: a historical cohort study

**DOI:** 10.1186/s12887-020-02325-0

**Published:** 2020-09-03

**Authors:** I. A. L. Bugter, L. C. E. Janssen, J. Dieleman, B. W. Kramer, P. Andriessen, H. J. Niemarkt

**Affiliations:** 1 Department of Neonatology, Máxima Medical Centre, De Run 4600, 5504 DB Veldhoven, The Netherlands; 2 Máxima Medical Centre Academy, Máxima Medical Centre, Veldhoven, the Netherlands; 3 Department of Pediatrics, Maastricht University Medical Centre, P Debyelaan 20, 6229 HX Maastricht, The Netherlands

**Keywords:** Preterm, Respiratory distress syndrome (RDS), Less invasive surfactant administration (LISA), Minimally invasive surfactant therapy (MIST), Surfactant, Avoidance of mechanical ventilation

## Abstract

**Background:**

In preterm infants with Respiratory Distress Syndrome (RDS), Less Invasive Surfactant Administration (LISA) has been established to reduce the need of mechanical ventilation and might improve survival rates without bronchopulmonary dysplasia. The aim of this study was to investigate whether NICU care has changed after introduction of less invasive surfactant administration (LISA), with regard to diagnostic and therapeutic procedures in the first week of life.

**Methods:**

Infants with gestational age < 32 weeks who received surfactant by LISA (June 2014 – December 2017, *n* = 169) were retrospectively compared to infants who received surfactant after intubation (January 2012 – May 2014, *n* = 155). Local protocols on indication for surfactant, early onset sepsis, blood transfusions and enteral feeding did not change between both study periods. Besides, as secondary outcome complications of prematurity were compared. Data was collected from electronic patient files and compared by univariate analysis through Students T-test, Mann Whitney-U test, Pearson Chi-Square test or Linear by Linear Association.

**Results:**

All baseline characteristics of both groups were comparable. Compared to controls, LISA patients received a higher total surfactant dose (208 vs.160 mg/kg; *p* < 0.001), required redosing more frequently (32.5% vs. 21.3%; *p* = 0.023), but needed less mechanical ventilation (35.5% vs. 76.8%; *p* < 0.001). After LISA, infants underwent fewer X-rays (1.0 vs. 3.0, *p* < 0.001), blood gas examinations (3.0 vs. 5.0, *p* < 0.001), less inotropic drugs (9.5% vs. 18.1%; *p* = 0.024), blood transfusions (24.9% vs. 41.9%, *p* = 0.003) and had shorter duration of antibiotic therapy for suspected early onset sepsis (3.0 vs. 5.0 days, *p* < 0.001). Moreover, enteral feeding was advanced faster (120 vs. 100 mL/kg/d, *p* = 0.048) at day seven. There were no differences in complications of prematurity.

**Conclusion:**

The introduction of LISA is associated with significantly fewer diagnostic and therapeutic procedures in the first week of life, which emphasizes the beneficial effects of LISA.

## Background

Non-invasive strategies in neonatal care of preterm infants are becoming increasingly important. However, a significant proportion of preterm infants with respiratory distress syndrome (RDS) fails non-invasive respiratory support alone and need exogenous surfactant (SF) replacement therapy [1]. In order to avoid intubation and mechanical ventilation, alternative approaches are developed. The most applied method of those is the INtubate-SURfactant-Extubate (INSURE) technique. However, with this technique a large proportion of infants still failed to be weaned of the ventilator immediately after surfactant treatment [2]. Other methods are still being studied, for example the INtubate-RECruite-SURfactant-Extubate (IN-REC-SUR-E) technique, SF via laryngeal mask or nebulised SF [3, 4]. A new recently investigated technique is called less invasive surfactant administration (LISA), in which SF is administered via a thin catheter to spontaneously breathing infants on nasal continuous positive airway pressure (CPAP) [5]. Recent studies show that LISA might be associated with higher survival rates without bronchopulmonary dysplasia (BPD) and might lead to fewer other complications of preterm birth, such as severe IVH, when compared to endotracheal SF administration after intubation [6–9].

However, when we introduced LISA to our level III NICU, we observed that LISA did not only affect the need of mechanical ventilation. It also changed the way we care for these infants in early life. This includes diagnostic and therapeutic procedures, which are potentially stressful or harmful for the infant, which is in line with our focus on family centred care. In our single room maternity NICU, mother-child bonding and the avoidance of stressful events are highly valuated.

We thus hypothesized that LISA, by preventing intubation and mechanical ventilation, is associated with a reduction in diagnostic and therapeutic procedures in early life. Therefore, the aim of our study was to quantify our experience and investigate if and how care has changed since the implementation of LISA, regarding diagnostic procedures and treatment in the first week of life. As a secondary outcome, complications of prematurity were compared. It is necessary and relevant to obtain objective and quantitative insight in those potential differences in practice, since they may lead to a change in the stress burden for the infant and in healthcare costs.

## Methods

A single-centre, historical cohort study was conducted in a level III NICU in The Netherlands. This study was approved by the local ethical board. Data was retrospectively extracted from electronic patient files and subsequently anonymised for analysis.

### Study population and data collection

**Fig. 1:**
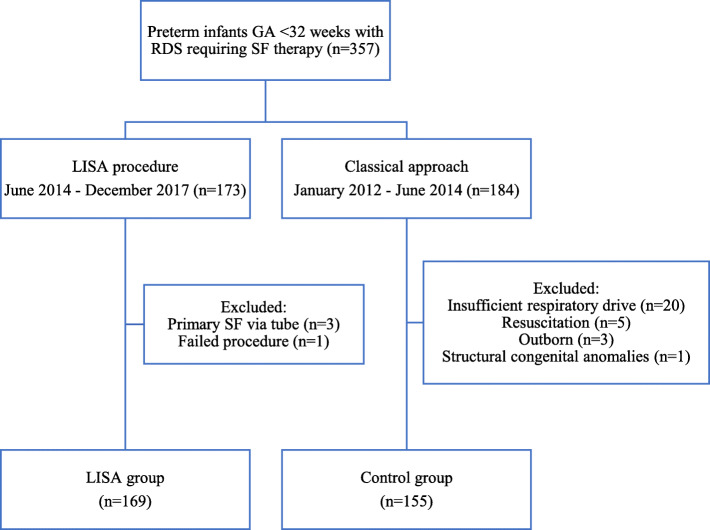
Flowchart summarising the patient selection procedure

All preterm infants born between 24 + 0 and 31 + 6 weeks of gestation, who received SF therapy for RDS between January 2012 and December 2017, were eligible. The study population was divided into two groups as shown in Fig. 1. The control group consisted of patients who received SF after endotracheal intubation followed by mechanical ventilation (January 2012–May 2014). The intervention group consisted of patients receiving SF via LISA (June 2014 – December 2017). All patients in both study groups received CPAP 6 cm H_2_O support directly after birth with a T-piece resuscitator in the delivery room. Infants were only directly intubated in the delivery room in case of severe persistent apnoea. Infants in the control group who showed insufficient respiratory drive and therefore would not have qualified for LISA during the intervention period, were excluded from analysis. Patients were also excluded if they were out born and intubated before transportation to our NICU centre or if structural congenital anomalies were present. Demographic data, Apgar scores, umbilical-pH, FiO_2_ before surfactant (as measure of RDS severity) were collected. Local protocols on indication for surfactant, Early Onset Sepsis (EOS), blood transfusions and enteral feeding did not change between both study periods.

### SF treatment

In both study periods (control and LISA), the aim was to treat with early rescue SF therapy conform the European Consensus Guidelines on the Management of Respiratory Distress Syndrome [10]. All patients received SF if they were on nasal CPAP of 6 cm H_2_O and their fraction of inspired oxygen (FiO_2_) was above 0.30. The maximum time window for SF replacement treatment was 72 h after birth. Patients were treated with 100–200 mg/kg Poractant alfa (Chiesi Pharmaceuticals, Parma, Italy).

Patients in LISA group were treated according to the Dutch LISA guideline. Infants received atropine (5 micrograms/kg) and a loading dose of intravenous caffeine (20 mg/kg) before LISA. No sedative medication was used prior to the procedure. A 4–5 French umbilical catheter or LISAcath® (Chiesi Pharmaceuticals, Parma, Italy) was placed below the vocal cords under direct laryngoscopy, with or without the use of Magill forceps. The laryngoscope was removed, and SF was subsequently administered in 2 to 3 min. During the procedure, nasal CPAP therapy at 6–8 cm H_2_O was continued.

The patients in the control group were intubated after administration of atropine, fentanyl and when necessary rocuronium. Infants were extubated as soon as they met the predefined extubation criteria. Those include a sufficient respiratory drive, low positive inspiratory pressure on volume targeted ventilation (< 18 cm H_2_O, with tidal volumes of 4–5 ml/kg) and low FiO_2_ (< 0.30). No medication was administered to antagonize the relatively short-acting sedatives that were used.

### Outcome measurements

Outcome variables were divided into two different groups: diagnostic procedures and treatment in the first week of life. As secondary outcomes, complications of prematurity were compared.

Diagnostic procedures were related to the number of X-rays, blood sample analyses and blood cultures for suspected EOS.

Outcomes regarding treatment included details on SF treatment, respiratory support, drug prescriptions, blood transfusions, enteral feeding, need for central catheters and phototherapy. SF therapy was specified with the total used dose of Poractant alfa and repeated procedures via LISA or endotracheal tube. The need for mechanical ventilation for at least 12 h and the absolute days on mechanical ventilation were reported. Information on the use of morphine, midazolam, inotropes, insulin, hydrocortisone and antibiotics was collected. Patent Ductus Arteriosus (PDA) treatment course included the number of courses of ibuprofen or indomethacin during NICU admission. The number of patients who underwent surgical ligation of a PDA was reported as well. Erythrocyte and thrombocyte transfusions reflect on the number of given transfusions in the first week of life. Enteral feeding was recorded as the amount of enteral nutrition given on day five and day seven in millilitres per kilogram per day.

Relevant complications of prematurity, which were reliable to find retrospectively, were supplementary oxygen on day 28 in survivors, surgical ligation of PDA, abdominal surgery on account of necrotizing enterocolitis (NEC) or spontaneous intestinal perforation (SIP), intraventricular haemorrhage (IVH) grade 3–4, pneumothorax after SF treatment, laser surgery because of retinopathy of prematurity (ROP) and mortality during NICU admission.

### Statistical analysis

The statistical analysis was performed using SPSS V.22. Continuous variables were compared by Students T-test or Mann Whitney-U test, depending on distribution. Outcomes were presented as mean with standard deviation (SD) when normally distributed and as median with interquartile range (IQR) when skewed. Nominal and ordinal variables were compared by Pearson Chi-Square test and Linear by Linear Association, respectively. Considering nominal variables, when the expected count of more than 20% of the cells was fewer than 5, Fisher’s Exact test was used. Those outcomes were presented as frequencies with percentages. Statistical significance was accepted at a two-sided *p*-value of fewer than 0.05.

## Results

### Study population and baseline characteristics

In the study cohort, *n* = 169 infants received exogenous SF therapy via LISA between June 2014 and December 2017. Control group consisted of *n* = 155 infants, who received SF therapy via endotracheal tube from January 2012 till June 2014. Baseline characteristics were compared and are shown in Table 1. The study groups were comparable with regard to gestational age, birth weight, sex, Apgar score, umbilical cord pH, maximal FiO_2_ before SF therapy and administration of antenatal steroids. Gestational age was also compared per category of antenatal steroids. This analysis showed no significant differences as well.

**Table 1:** Baseline characteristics of the study population

	LISA (***n*** = 169)	Control (***n*** = 155)	***p***-value
GA (weeks + days), mean (SD in days)	28 + 0 (14)	28 + 1 (14)	0.52
GA categories			0.26
24–25 + 6 weeks	29 (17.2%)	21 (13.5%)	
26–27 + 6 weeks	58 (34.3%)	50 (32.3%)	
28–31 + 6 weeks	82 (48.5%)	84 (54.2%)	
Birth weight (grams), mean (SD)	1092 (341)	1130 (350)	0.33
Birth weight categories			0.44
< 750 g	29 (17.2%)	22 (14.2%)	
750 - 999 g	50 (29.6%)	43 (27.7%)	
1000 - 1249 g	34 (20.1%)	36 (23.2%)	
≥ 1250 g	56 (33.1%)	54 (34.8%)	
Male sex	94 (55.6%)	84 (54.2%)	0.80
Antenatal corticosteroids			0.82
None	8 (4.7%)	9 (5.8%)	
Not completed	53 (31.4%)	43 (27.7%)	
Completed	108 (63.9%)	103 (66.5%)	
GA (weeks+days) per category of corticosteroids, mean (SD)			
None	27 + 3 (13)	28 + 0 (10)	0.44
Not completed	28 + 2 (14)	28 + 5 (15)	0.21
Completed	27 + 6 (15)	27 + 6 (14)	0.83
Apgar, median (IQR)			
1 min	6 (4.0–6.5)	5 (4–7)	0.89
5 min	7 (6–8)	7 (6–8)	0.70
Umbilical cord pH, median (IQR)	7.30 (7.26–7.34)	7.30 (7.24–7.34)	0.48
Maximal FiO_2_ before SF, median (IQR)	35 (30–45)	40 (33.75–50)	0.15
Time birth-SF (hours), median (IQR)	2 (1.5–3.75)	2.5 (2–5)	0.001*

*GA* Gestational age, *LISA* Less Invasive Surfactant Administration, *GA* Gestational age

### Diagnostic procedures

Diagnostic features of the study population are shown in Table 2. Patients treated with LISA underwent fewer X-rays compared to controls (median 1 vs. 3, *p* < 0.001), especially with regard to chest X-rays (median 1.0 vs. 2.0, *p* < 0.001). Besides, the absolute number of collected blood gas samples was reduced in the first week of life, showing almost twice as much collections in the controls (median 3 vs. 5, *p* < 0.001). In the LISA group, 68.0% of patients were treated for suspected EOS, compared to 77.9% in control group (*p* = 0.046). No differences between the study groups were found in the number of other blood samples for laboratory in the first week.

**Table 2:** Diagnostic features of LISA group compared to control group

	LISA (***n*** = 169)	Control (***n*** = 155)	***p***-value
X-rays, median (IQR)			
Chest 1st week	1.0 (0–2)	2.0 (1–4)	< 0.001*
All 1st week	1.0 (0–3)	3.0 (1–4)	< 0.001*
Laboratory tests in 1st week, median (IQR)			
Blood gas	3.0 (1–6)	5.0 (3–11)	< 0.001*
Haematology	6.0 (4–8)	5.0 (4–7)	0.108
Chemistry	8.0 (7–9)	8.0 (7–10)	0.481
Blood culture for suspected EOS	115 (68.0%)	120 (77.9%)	0.046*

*shows significance (*p* < 0.05)

### Treatment

Treatment features for both groups are shown in Table 3. Patients treated with LISA needed more than one SF dose in 32.5% of cases, compared to 21.3% in the control group (*p* = 0.023). In the LISA treated infants, 20.7% received a second dose of SF via an endotracheal tube after intubation. Initial and total dose of SF were higher in LISA group and showed a difference between the study groups of 41 and 48 mg/kg, respectively (*p* < 0.001). In infants treated with LISA, 38.5 and 35.5% of patients were intubated in the first week and needed mechanical ventilation for at least 12 h, respectively. The latter was needed in 78.8% of patients in the control group (*p* < 0.001). Control patients were treated with inotropic drugs roughly twice as much, compared to LISA patients (18.1% vs. 9.5%, *p* = 0.024). Duration of empirical antibiotic treatment for suspected (blood culture negative) EOS was longer in control patients (median 5.0 vs. 3.0 days, *p* < 0.001). Erythrocyte transfusions were administered more frequently in the control group compared to LISA group (41.9% vs. 24.9%, *p* = 0.001). The increase of enteral feeding was faster in the LISA group compared to controls, with a larger volume on day seven (120 vs. 100 mL/kg, *p* = 0.048). In the first week of life, LISA patients received an umbilical catheter in 37.9% of the cases, compared to 51.6% of control patients (*p* = 0.013). No differences between the groups were found considering PDA treatment courses, thrombocyte transfusions or days on phototherapy in the first week of life.

**Table 3:** Treatment features of LISA group compared to control group in the first week of life

	LISA (***n*** = 169)	Control (***n*** = 155)	***p***-value
Surfactant			
Multiple gifts	55 (32.5%)	33 (21.3%)	0.023*
≥ 1 endotracheal tube gift	35 (20.7%)	155 (100%)	< 0.001*
Initial dose (mg/kg), median (IQR)	189 (162–207)	148 (126–174)	< 0.001*
Total dose (mg/kg), median (IQR)	208 (178–329)	160 (129–198)	< 0.001*
Respiratory support			
Intubation	65 (38.5%)	155 (100%)	< 0.001*
MV 12 h	60 (35.5%)	119 (76.8%)	< 0.001*
MV (days), median (IQR)	0.0 (0–1)	1.0 (1–3)	< 0.001*
Drug prescriptions			
Morphine	20 (11.8%)	23 (14.8%)	0.426
Midazolam	3 (1.8%)	1 (0.6%)	0.358
Inotropes	16 (9.5%)	28 (18.1%)	0.024*
Insulin	8 (4.7%)	13 (8.4%)	0.182
Antibiotics for suspected EOS (days), median (IQR)	3 (2–6)	5 (3–7)	< 0.001*
PDA treatment courses			0.658
None	91 (53.8%)	84 (54.2%)	
1	46 (27.2%)	35 (22.6%)	
> 1	32 (18.9%)	36 (23.2%)	
Erythrocyte transfusions			0.003*
None	127 (75.1%)	90 (58.1%)	
1	23 (13.6%)	37 (23.9%)	
> 1	19 (11.2%)	28 (18.1%)	
Thrombocyte transfusions			0.458
None	153 (90.5%)	136 (87.7%)	
1	10 (5.9%)	12 (7.7%)	
> 1	6 (3.6%)	7 (4.5%)	
Enteral feeding (mL/kg), median (IQR)			
Day 5	88 (50–120)	60 (40–120)	0.051
Day 7	120 (80–160)	100 (60–140)	0.048*
Umbilical catheter	64 (37.9%)	80 (51.6%)	0.013*
Phototherapy (days), mean (SD)	2.1 (1.8)	2.1 (1.6)	0.826

*shows significance (*p* < 0.05)

*MV 12 h* Mechanical ventilation for more than 12 h, *PDA* Patent ductus arteriosus

### Complications of prematurity

As shown in Table 4, no statistically significant differences were found in complications of prematurity.

**Table 4:** Complications of LISA group compared to control group

	LISA (***n*** = 169)	Control (***n*** = 155)	***p***-value
Supplementary oxygen at day 28 in survivors	79 (49.7%)	63 (45.3%)	0.452
Surgical ligation of PDA	9 (5.3%)	15 (9.7%)	0.135
Abdominal surgery	9 (5.3%)	7 (4.5%)	0.737
IVH grade 3–4	11 (6.5%)	13 (8.4%)	0.519
ROP laser surgery	6 (3.6%)	4 (2.6%)	0.614
Pneumothorax	3 (1.8%)	6 (3.9%)	0.320
Mortality during NICU admission	14 (8.3%)	16 (10.3%)	0.527

*shows significance (*p* < 0.05)

*PDA* Patent ductus arteriosus, *IVH* Intraventricular hemorrhage, *ROP* Retinopathy of prematurity

## Discussion

In this study, we investigated the effects of LISA introduction in a tertiary NICU on several diagnostic and therapeutic procedures in the first week of life. We found that LISA implementation was associated with remarkable changes in neonatal practice. To the best of our knowledge this study is the first that provides insight into changes in NICU practice following LISA implementation, compared to classical SF administration. Previous studies in this field have only focused on variables related to respiratory support, pulmonary outcomes and complications [3, 4, 11].

Both groups were similar regarding baseline characteristics. Postmenstrual age birthweight and FiO2 before SF treatment not significantly different between groups. The threshold for surfactant treatment was identical in both groups. Therefore the groups comparable to each other.

With respect to diagnostic procedures, LISA was associated with fewer X-rays. This is desirable, since all ionizing irradiation will increase the cumulative risk for radiation induced tumorigenesis [12]. This finding might be explained because X-rays were mostly performed to verify endotracheal tube position before administering SF. We did not routinely perform X-rays to establish diagnosis of RDS, but relied on the clinical presentation. However, when diagnosis of RDS was questioned, or when pneumothorax or atelectasis was suspected, a chest X-ray was performed as well.

In LISA patients, fewer blood gas analyses and collections were performed. We have used throughout the entire study period we have used volume-targeted ventilation and performed non-invasive carbon dioxide monitoring (transcutaneous and/or end-tidal) in our NICU centre. Fewer blood gas sampling leads to a reduction in blood loss and skin breaking procedures. We expect that this difference in blood gas sampling will even be larger in centres who do not use volume-targeted ventilation and non-invasive carbon dioxide monitoring [13–15]. The saved resources on blood-gas measurements are a direct pharmaco-economic saving. Unfortunately, we have no numbers to quantify this saving in the present study.

Despite the higher initial dose of administered SF in LISA group, those patients needed redosing more often. Both the higher initial dose and redosing more frequently might be due to the knowledge clinicians gained on SF spill, loss of surfactant in de application device and a different pulmonary distribution of SF, which are reported to contribute to a higher required dose of SF for LISA patients [16, 17]. In addition, a SF dose of fewer than 200 mg/kg is associated with LISA failure. This difference therefore possibly contributes to the higher rate of multiple administered gifts of SF in LISA group, since other risk factors for LISA failure (such as male gender) were comparable between the study groups [18]. Regarding respiratory support, reduction in the need for mechanical ventilation when using LISA is a well-established finding, which is reconfirmed in our results [6]. However, we did not find a significant difference in short term respiratory outcome.

An interesting and important finding is the lower incidence and shorter duration of antibiotic treatment for suspected EOS in patients treated with LISA. The Dutch protocol on EOS, which was based on the NICE guideline, mandates a critical review before the start and continuation of antibiotic treatment after 48 h of therapy. This protocol did not change during the study period. There was neither a significant difference in culture-proven EOS between LISA patients and controls (3.0% vs 1.3%, *p* = 0.302).

The adverse effects of antibiotics were already known before the study period. Antibiotic use in early life is known to disturb microbiota development in preterm infants [19]. Besides, reduction of unnecessary antibiotic treatment is of importance, as each day of prolonged early use is associated with an increased risk for late-onset sepsis (LOS), NEC or death [20]. However, reducing antibiotic use in daily NICU practice is still challenging. The decision to start and stop antibiotic therapy for suspected EOS is partly based on the assessment of the attending physician of the clinical condition of the infant and therefore subjective [11]. A preterm infant on non-invasive respiratory support is likely to be considered in clinically better condition than a mechanical ventilated infant. Therefore, the introduction of LISA, leaving less infants on mechanical ventilation, may have helped in deciding not to start or early discontinue antibiotic treatment for suspected EOS.

A large difference was found in the need for inotropic support between both study groups, with a significant lower need in LISA patients. This may be a logical finding, as sedatives and analgesics, which are both frequently used with endotracheal intubation and mechanical ventilation, often induce hypotension as side effect [21–23]. Moreover, mechanical ventilation itself might more often lead to hypotension as well [24]. During the study period no delayed cord clamping was performed in our NICU centre, which therefore did not affect our observations. The early use of inotropes is associated with severe IVH and increased mortality [25]. Therefore, we hypothesise that reduction in the use of inotropes may explain the lower rate of severe IVH in LISA treated infants in the NINSAPP study [26]. Furthermore, LISA was associated with a decrease of erythrocyte transfusions. The most logical explanation is the use of lower thresholds for erythrocyte transfusion in ventilated infants compared to non-ventilated infants in the Dutch protocol for blood transfusions [27]. Studies suggest that early erythrocyte transfusions are associated with an increased risk of severe IVH, ROP and mortality [28–30]. Therefore, the LISA associated reduction of may be of benefit for the patient.

Based on our findings, implementation of LISA may help in advancing enteral feeding, which resulted in higher amounts of enteral feeding at day 7 in LISA patients. According to our local feeding protocol, which did not change between the two study periods, preterm infants < 27 weeks of gestation or infants with birth weight < 3rd percentile received trophic feeding for 2 to 3 days. Hereafter, feeding was increased with 20 mL/kg/day if well tolerated (guided by clinical appearance, abdominal distension and/or tenderness, emesis, and large gastric residuals). The remaining infants started with enteral feeding immediately. Enteral feeding might be tolerated better by non-ventilated infants and may be explained partly by the lower but not significant difference in morphine administration. Moreover, the attending clinician could be less inclined to withhold feeding advancement in non-ventilated infants, who are deemed in a better clinical condition more often. Aggressive feeding strategies may reduce the risk of late-onset sepsis, without increasing the risk of NEC [31, 32]. Therefore, we think that this difference in enteral feeding advancement may be beneficial for those infants. We hypothesize that this finding may have contributed to the observation that 55.9% of infants in LISA group, compared to 32.0% in control group, having regained birth weight in the first week of life (*p* < 0.001).

In this study, we did not find statistically significant differences in complications of prematurity between our groups. We think this is due to our small study groups. It would be interesting to have data on our primary outcomes from studies in which those differences in complications were found. Unfortunately, we could not provide data on BPD incidence and severity, as a large proportion infants were transferred to other hospitals before the postmenstrual age of 36 weeks and no oxygen reduction test was performed. Therefore we were restricted to report only on supplementary oxygen at 28 days as proxy diagnosis of developing BPD.

Overall, we found several changes in NICU practices in early life, which may have beneficial effects on short-term and long-term outcomes of preterm infants with RDS treated with LISA and lower use of healthcare resources. In this study, we did not focus on the long term outcome, as groups were considered too small to find statistically significant effects. We think this is the reason we found no difference in short-term outcomes as well. However, in future meta-analyses of studies with LISA including large groups of infants, effects of changes in NICU care aspects on these outcomes may be of interest. Besides, this study was performed on a single room maternity care with a special interest in family centred care. We consider a reduction in skin breaking procedures and fewer diagnostic procedures, which lead to less radiation load, as beneficial for these infants. Moreover, the presence of umbilical catheters, inotropic medication and respiratory instability are often barriers for Kangaroo care. The significant reduction in these interventions, will make it easier to start with Kangaroo care early in life, which, in our view, is of importance for these infants and their parents. Regrettably, we did not have objective data about the amount and duration of Kangaroo care in the first week of life. At last, the reduction in diagnostic and therapeutic procedures may reduce costs, although this may not weigh up against the costs of higher amount of administered SF in the LISA patients. However, this should be investigated in an extensive cost-effectiveness study.

A main strength of this study is the use of strict local protocols in our NICU, which reduces the variation of care among the attending neonatologist and nurses. Our protocols regarding enteral feeding, EOS or blood transfusions did not change during the study period. Another strength is that data was collected from electronic patient files, providing complete and reliable data.

However, there are several limitations that need to be acknowledged. Most importantly, this is a historical cohort study, with evident risks of confounding. Although protocols on the studied parameters did not change, implicit changes in NICU care, regardless of LISA implementation may have taken place which confounded results. However, baseline characteristics (gestational age, birth weight, sex, antenatal steroids, Apgar score, umbilical cord pH) and RDS severity (determined as FiO_2_ before surfactant) were similar between both study groups. Therefore, in our opinion, the observed significant differences in diagnostic and treatment procedures are largely explained by the introduction of LISA. However, in order to confirm our observations, the changes in NICU care associated with LISA should may be subject of investigation in future randomized trials or a prospective cost-effectiveness study. Furthermore, our findings may not be extrapolated easily to other NICU centres implementing LISA, as this was a single centre study in the Dutch healthcare setting. However, our study provides items that other centres may evaluate before and after the introduction of LISA in order to assess the effects on care, outcome and resource allocation.

## Conclusion

In conclusion, our findings demonstrate that introduction of LISA, as one of the minimally invasive surfactant procedures, has contributed to a change in practice towards fewer diagnostic and therapeutic interventions in preterm infants with RDS. We hypothesize that the reduction in diagnostic and therapeutic interventions in these vulnerable infants may explain the fewer complications after LISA in larger study groups and provides more opportunities for Kangaroo care. In addition, the allocation of healthcare resources changed, with more surfactant being administered with LISA but less x-rays and blood gas analyses being performed. These findings emphasize the beneficial effects of using LISA as the primary management of RDS with exogenous SF requirement.

## Data Availability

The datasets used and/or analysed during the current study are available from the corresponding author on reasonable request.

## Abbreviations

CPAP: Continuous positive airway pressure
GA: Gestational age
EOS: Early Onset Sepsis
IQR: Interquartile range
IVH: Intraventricular haemorrhage
LISA: Less invasive surfactant administration
NEC: Necrotizing enterocolitis
NICU: Neonatal intensive care unit
PDA: Patent ductus arteriosus
RDS: Respiratory distress syndrome
ROP: Retinopathy of prematurity
SD: Standard deviation
SF: Surfactant
